# Complete Genome Sequence of the Acetylene-Fermenting *Pelobacter* sp. Strain SFB93

**DOI:** 10.1128/genomeA.01573-16

**Published:** 2017-02-09

**Authors:** John M. Sutton, Shaun M. Baesman, Janna L. Fierst, Amisha T. Poret-Peterson, Ronald S. Oremland, Darren S. Dunlap, Denise M. Akob

**Affiliations:** a Department of Biological Sciences, The University of Alabama, Tuscaloosa, Alabama, USA; b U.S. Geological Survey, National Research Program-Western Branch, Menlo Park, California, USA; c U.S. Geological Survey, National Research Program-Eastern Branch, Reston, Virginia, USA

## Abstract

Acetylene fermentation is a rare metabolism that was previously reported as being unique to *Pelobacter acetylenicus*. Here, we report the genome sequence of *Pelobacter* sp. strain SFB93, an acetylene-fermenting bacterium isolated from sediments collected in San Francisco Bay, CA.

## GENOME ANNOUNCEMENT

Acetylene (C_2_H_2_) fermentation is a unique metabolic pathway found in only three well-characterized strains within the *Pelobacter* genus (1–3). Currently, little is known about the regulation and evolution of acetylene metabolism in nature. Here, we present the complete genome sequence for *Pelobacter* sp. strain SFB93 (2, 3).

*Pelobacter* sp. SFB93 was isolated from sediments collected from San Francisco Bay, CA, and grown in the anaerobic bicarbonate-buffered bay water medium (ABW) (3). The genome of *Pelobacter* sp. SFB93 was sequenced at the University of California at Davis Genome Center (Davis, CA, USA) on a Pacific Biosystems (PacBio) single-molecule real-time (SMRT) RSII instrument (Pacific Biosciences of California, Inc., Menlo Park, CA). Briefly, DNA was extracted from cultures with the DNeasy blood and tissue kit (Qiagen, Valencia, CA, USA), according to the manufacturer’s instructions but modifying the protocol by inverting in place of vortexing to limit DNA shearing. DNA from several extractions was concentrated with either a Power Clean Pro kit (Mo Bio Laboratories, Inc., Carlsbad, CA, USA) or a Zymo Spin genomic DNA (gDNA) concentrator (Zymo Research Corporation, Irvine, CA, USA). DNA extracts were sent to the Davis Genome Center, and sequencing libraries were prepared with 10-kb inserts. Sequencing on a single SMRT cell with C4 chemistry produced 63,938 sequence reads with an *N*_50_ read length of 18,803 bp.

These sequence reads were subsequently assembled using the Hierarchical Genome Assembly Process (HGAP; Pacific Biosciences of California, Inc., Menlo Park, CA) (4), including polishing with Quiver. *Pelobacter* sp. SFB93 contains a single 3,218,469-bp circular chromosome with 53.4% G+C content (87× average coverage). The Prokaryotic Genome Annotation Pipeline (PGAP) from the National Center for Biotechnology Information (NCBI) was used for functional annotation (5). The *Pelobacter* sp. SFB93 genome contained 2,915 genes and 2,851 coding sequences (CDSs). Functional annotation identified nine rRNAs, 50 tRNAs, five noncoding RNAs, and 74 pseudogenes. No clustered regularly interspaced short palindromic repeat (CRISPR) genes were annotated in *Pelobacter* sp. SFB93.

The fermentation of acetylene to acetate and ethanol requires five functional enzymes (acetylene hydratase [AH], aldehyde dehydrogenase, alcohol dehydrogenase, phosphate acetyltransferase, and acetate kinase). Gene sequences that code for the five enzymes involved in acetylene fermentation were annotated in this genome. *Pelobacter* sp. SFB93 has two copies of the* ahy* gene that codes for a functional AH. The two copies are 96.9% identical to one another on the amino acid level. Alignment to *Pelobacter acetylenicus* type strain DSM3246 showed 88.4% and 90.4% amino acid sequence similarity between the *ahy* sequences encoded in the presented genome and the single copy found in *P. acetylenicus* type strain DSM3246 (GenBank accession no. AF518725.1). Comparative genomic analyses between *Pelobacter* sp. SFB93 and the acetylene-fermenting *P. acetylenicus* strains DSM3246 and DSM3247 may provide insight into the regulation and evolution of acetylene metabolism.

### Accession number(s).

Genomes are available from the NCBI GenBank database under BioProject PRJNA319824 and GenBank accession no. CP015519 (*Pelobacter* sp. SFB93).
